# SARS-CoV-2 IgG Surveillance in Asymptomatic Blood Donors and Health Workers

**DOI:** 10.1155/2021/2404170

**Published:** 2021-12-29

**Authors:** Tulika Chandra, Devisha Agarwal, D. Himanshu, Mallika Agarwal, Bipin Puri

**Affiliations:** ^1^Department of Transfusion Medicine, King George Medical University, Lucknow, India; ^2^Department of Otolaryngology, King George Medical University, Lucknow, India; ^3^Department of Medicine, King George Medical University, Lucknow, India; ^4^Era Medical College, Lucknow, India; ^5^King George Medical University, Lucknow, India

## Abstract

**Materials and Methods:**

2085 blood donors were allowed to donate blood only after fulfilling all the criteria laid down by the FDA of India with additional history of excluding COVID-19 suspects. IgG antibody testing was performed by chemiluminescence, and results were noted along with their reactive status. Their reactive status was analyzed with donor information to get an idea of the risk parameters for COVID-19. Medical healthcare workers in whom the study was carried out were 560, out of which 114 had worked in COVID-19 duties and 446 had worked in non-COVID-19 emergencies areas. COVID-19 area duties were further subdivided into triage, holding area, isolation, and COVID-19-related duties. The samples were run on architect i2000 and evaluated for their plasma immunoglobulin G.

**Results:**

Amongst the asymptomatic blood donors, 1.9% was found to be COVID-19 IgG antibody positive. It was observed that maximum COVID-19 IgG positivity (57.1%) was seen in the age group 18–29 years followed by 26.2% in the age group 30–39 years. Donors in the age group 40–49 years showed antibody positivity of 16.7%, and no antibody-positive donors were found above 50 years of age. COVID-19 IgG positivity was maximum in replacement donors (61.9%) followed by family donors (28.6%) and least involuntary donors (0.6%) Blood donors who showed high IgG positivity were mainly of labor class. Antibody IgG testing on medical healthcare workers showed 2.3% positivity. The healthcare workers who were posted in COVID-19 duties showed 4.8% positivity in the holding area (waiting area with the treatment of patients till their RT PCR report comes) and 5.7% in other COVID-19 areas related to laboratory work. Healthcare workers doing duties in COVID-19 areas showed 2.7% positivity, while those doing duties in non-COVID-19 emergency areas showed a positivity of 2.2%.

**Conclusion:**

Our study shows that the prevalence of detectable antibodies was low in the general population in India and many patients were asymptomatic as seen in the blood donors, especially the labor class. Maximum exposure was present in young healthy males of labor class who remained asymptomatic. The healthcare workers were more exposed to COVID-19 as compared to the general population probably due to lack of precaution and awareness. Those doing non-COVID-19 duties were also exposed appreciably and needed to take all the precautions required for COVID-19 duties.

## 1. Background and Objectives

SARS-CoV-2 virus has caused a global pandemic as declared by the WHO in March 2020. In India, the first case was reported in Kerala on the 30th of January, and since then, many states are active but some are showing flattening [1].

Reverse Transcriptase Polymerase Chain Reaction (RT-PCR) is being considered as the gold standard technique that confirms the presence of COVID-19 [2]. However, testing of specimens from multiple sites may improve the sensitivity and reduce false-negative test results [3]. Due to the high infectivity rate and a majority of patients being asymptomatic, widespread serological screening was required. Memory antibody (IgG type) assessment is useful as many healthy individuals in the epidemic area may be asymptomatic but infected and possess a threat for further spread of the virus. Monitoring IgG will in turn help us for guiding future decisions on the right time to relax social distancing measures and minimize possible epidemic outbreaks [4]. The role of SARS-CoV-2 IgG antibodies, verified by PCR, has shown positive results for confirming the infection [5]. Both SARS-CoV and MERS-CoV infections were believed to originate in bats, and these were transmitted directly to humans [6]. Following the seroprevalence testing in healthy blood donors, we can monitor the spread of the virus among healthy people and, thus, will eventually lead to implementing strategies to reduce the spread. Evidence is also emerging that monocyte/macrophage dysfunction may be central to the immunopathology [7] and that the functional characteristics of antibodies to SARS‐CoV‐2 spike protein (SP) might be a determinant of disease outcome. Antibody response against enveloped viruses such as SARS‐CoV‐2 comprises immunoglobulin (Ig) M, IgG3, IgG1, and IgA antibodies to glycoproteins of the virus envelope and nucleoproteins (NP, internal to the envelope). IgG (IgG3 and IgG1) antibodies against virus envelope glycoproteins have many functional characteristics that make up the most efficacious antibody response against viruses, as illustrated by human immunodeficiency virus- (HIV-) 1 infection [8]. Healthcare workers are the frontline workforces who provide care to both suspected as well as confirmed COVID-19 cases. Thus, they are at a higher risk of acquiring the disease, and if they are infected, they possess a serious risk for vulnerable patients as well as fellow healthcare workers. Thus, a need was felt to conduct a study to assess the IgG antibody status in healthcare workers differentiating those who were in COVID-19 and others in non-COVID-19 emergency duties during this pandemic. Antibodies in healthcare workers will help in maximizing our safe manpower resources and avoid unnecessary quarantine. Comparison with the general population which was represented by asymptomatic blood donors was carried out to assess the prevalence of antibodies in the population. The objective of the study was that, during the time of pandemics and panic amongst the general population and the government, we should be able to provide data regarding the safety measures of our medical staff and the development of antibodies in the population. A material and method setting study was carried out by the Department of Transfusion Medicine, King George's Medical University, Lucknow, in June 2020.It was carried out in 2121 asymptomatic blood donors and 560 medical healthcare workers working in COVID-19 and also non-COVID-19 emergency duties. The work was a part of university policy, and in order to screen its healthcare workers and blood donors for COVID-19 antibodies, adequate precautions and decisions could be taken accordingly. The department of transfusion medicine has one of the biggest blood banks in the country and caters to donors all over the state of Uttar Pradesh and, thus, represents a huge proportion of the asymptomatic population of the state of Northern India. King George's Medical University is the largest medical university in the country and is giving both COVID-19 and non-COVID-19 care to the patients. Sample collection: blood donors were allowed to donate blood only after fulfilling all the criteria laid down by the FDA of India with additional history of excluding COVID-19 suspects. This included contact history with any COVID-19-positive patient, recent travel history, and symptoms of COVID-19. Consent was taken regarding the screening of COVID-19 antibodies. They donated blood only after completing their fitness. In the blood units, apart from regular screening of HIV, hepatitis B, hepatitis C, malaria, and syphilis, testing for IgG antibodies for COVID-19 was also performed. It was carried out on 2 ml EDTA blood from which plasma was used after centrifugation and IgG antibodies testing was performed by chemiluminescence technology by SARS-CoV-2 IgG assay (Abbott Laboratories Inc. CLIA) on the Abbott ARCHITECT i system. The results of IgG antibodies (OD Values) were noted along with their reactive status. Their reactive status was analyzed with donor information to get an idea of the risk parameters for COVID-19. Medical healthcare workers in whom the study was carried out were 560. They included doctors, technicians, staff nurses, attendants, sweepers, and clerks and were randomly selected. Out of them, 114 had worked in COVID-19 duties and 446 had worked in non-COVID-19 emergencies areas. COVID-19 area duties were further subdivided into triage, holding area, isolation, and COVID-19-related duties. Healthcare workers working in different departments were screened to analyze the high-risk areas for COVID-19. 2 ml blood was collected from all the healthcare workers in EDTA vials. Blood was collected from healthcare workers from COVID-19 areas only after 14 days of quarantine which was the standing government policy. Non-COVID-19 workers were not quarantined; hence, blood was collected when they were on duty. The collection was carried out according to the guidelines of the university for understanding the spread of COVID-19 in the university which can be used later for duty rostering purposes. The samples were run on architect i2000 and evaluated for their Immunoglobulin G status. 24 samples that tested positive for antibodies were retested on a rapid card test Q Line Rapid for COVID-19 IgG to confirm its sensitivity and specificity.

## 2. Statistical Analysis

Chi-square tests were used for categorical variables for comparisons between groups. *p* ≤0.05 was considered statistically significant. Data collected were entered in the master chart and analyzed using the appropriate statistical procedure and SPSS software.

## 3. Results

Amongst the asymptomatic blood donors, 42 (1.9%) were found to be COVID-19 IgG antibody positive (Table 1). Amongst the 2085 samples, none of the 36 females were IgG positive in comparison to the remaining 2049 males in which 42 were positive for antibodies (Table 2). It was observed that the maximum COVID-19 IgG positivity (57.1%) was seen in the age group 18–29 years followed by 26.2% in the age group 30–39 years. Donors in the age group 40–49 years showed an antibody positivity of 16.7%, and no antibody-positive donors were found above 50 years of age (Table 3) COVID-19 IgG positivity was maximum in replacement donors (61.9%) followed by family donors (28.6%) and least involuntary donors (0.6%) (Table 4). Immune donors showed good hemoglobin with a mean of 14.05gm% (Table 5). Blood donors who showed high IgG positivity were mainly of labor class (Table 6). Antibody IgG testing on medical healthcare workers showed 2.3% positivity (Table 7). The healthcare workers who were posted in COVID-19 duties showed 4.8% positivity in the holding area (waiting area with the treatment of patients till their RT PCR report comes) and 5.7% in other COVID-19 areas related to laboratory work (Table 8). Healthcare workers doing duties in COVID-19 areas showed 2.7% positivity, while those doing duties in non-COVID-19 emergency areas showed a positivity of 2.2% (Table 9). Speciality-wise distribution of healthcare workers in different departments showed the highest antibody positivity in the respiratory ICU and prosthodontics department (100%) followed by trauma surgery (12.5%), blood bank (9.5%), radiodiagnosis (9.1%), plastic surgery (3.7%), urology (3.6%), general surgery (3.1%), and neurosurgery (2.6%) (Table 10).

## 4. Discussion

In our study, IgG status amongst blood donors was 1.9% which implied that no significant immunity had developed in the society even after two months of the pandemic. These donors belonged to all socioeconomic statuses and age groups between 18 and 65 years. A study published from the University Hospital, Eppendorf, presented data which showed that fewer than 1% of 914 tested regular blood donors had IgG antibodies against the COVID-19 virus [9]. Chang Le and Hou et al. reported that the SARS-CoV-2 seroprevalence among their blood donors was 2.29% (407/17,794, 95%CI: 2.08% to 2.52%) in Wuhan, 0.029% (2/6,810, 95%CI: 0.0081% to 0.11%) in Shenzhen, and 0.0074% (1/13,540, 95%CI: 0.0013% to 0.042%) in Shijiazhuang, respectively. The earliest emergence of SARS-CoV-2 seropositivity in blood donors was identified on January 20, 2020, in Wuhan [10]. Fischere et al. determined an overall low IgG seroprevalence of 0.91% (95% CI: 0.58–1.24) against SARS-CoV-2 in three German federal states. This was carried out in 3,186 regular blood donors in three German federal states between 9 March and 3 June 2020 [11].

Development of COVID-19 IgG antibodies in individuals indicated that they have been exposed to COVID-19 infection within the past two months, remained asymptomatic, and consequently developed antibodies against COVID-19. These individuals had been asymptomatic carriers of COVID-19 at some point in time. Bastian Fischere et al. also stated that all donors had undergone a complete medical examination before donation, they did not give any history of current or recent diseases and had no symptoms, and signs of infections were present such as fever or an increased leukocyte count. None of their seropositive blood donors for IgG antibodies reported a known positive medical history of SARS-CoV-2 infection [11]. COVID-19-positive antibodies were found in males only, which probably implied that females had not been exposed to COVID-19 due to the social and cultural structure of India in which majority of them remain mainly confined to their households while males tend to move out, mingle, and interact with groups. Hence, this increased the chances of exposure of males to COVID-19 during which they remained asymptomatic and, thus, resulted in the formation of antibodies.

The age group commonly which was infected and remained asymptomatic was 18–29 years. Young males are usually more social and move in the community despite restrictions of lockdown. This was probably the reason for their exposure. As maturity and responsibility increased, people were more aware of the seriousness of the pandemic and, hence, avoided the exposure which was absent above 50 years of age. Iversen et al. reported that “between April 15 and April 23, 2020, 29,884 healthcare workers were invited to take part in the study, out of which 29,117 (97%) participants were included, amongst which 28,792 (98%) provided SARS-CoV-2 antibody results. The mean age was 44·4 years (SD 12·6) years. 22,715 (78·9%) participants were female, and 6077 (21·1%) were male” [12]. Our study showed a different age group and gender because India is a country with a separate social structure.

Replacement donors were more exposed to COVID-19 as compared to voluntary donors, with the reason being probably the level of awareness. Voluntary donors are self-motivated about blood donation, and the awareness levels for COVID-19 are also more as they are aware of their social responsibilities and needs. The hemoglobin status of blood donors was 14.05 and showed appropriate hemoglobin which signified that COVID-19 had not been detrimental to their general health. COVID-19 was supposed to be a disease of the affluent class, but higher positivity in the labor class implied that may be due to better immunity, in India, due to multiple exposures to organisms, they were able to tolerate the COVID-19 asymptomatically in contrast to the affluent class. No published literature was obtained for the abovementioned facts because of the geographical variation of each country.

Rapid antibody kits showed higher specificity and can be used for screening, but negative results need further verification by chemiluminescence. In healthcare workers, the antibody was 2.3% which was higher than in the community signifying a higher exposure in a medical college. Iversen et al. also reported seroprevalence to be higher in healthcare workers than in blood donors (142 [3·04%] of 4672; risk ratio (RR) 1·33 [95% CI 1·12–1·58]; *p* < 0·001). They also said that seroprevalence was higher in male healthcare workers (331 [5·45%] of 6077) than in female healthcare workers (832 [3·66%] of 22 715; RR 1·49 [1·31–1·68]; *p* < 0·001). Frontline healthcare workers working in hospitals had a significantly higher seroprevalence (779 [4·55%] of 16 356) than healthcare workers in other settings (384 [3·29%] of 11 657; RR 1·38 [1·22–1·56]; *p* < 0·001). Healthcare workers working on dedicated COVID-19 wards (95 [7·19%] of 1321) had a significantly higher seroprevalence than other frontline healthcare workers working in hospitals (696 [4·35%] of 15 983; RR 1·65 [1·34–2·03]; *p* < 0·001). 622 [53·5%] of 1163 seropositive participants reported symptoms attributable to SARS-CoV-2. [13]. In India, in our study, healthcare workers employed in COVID-19 duties showed a positivity of 2.7% as compared to those in non-COVID-19 duties of 2.2%. The difference was negligible because in non-COVID-19 duties, we probably had many patients who did not show COVID-19-related symptoms or were false negatives, and hence, the healthcare workers whose level of safety and precautions were not to the appropriate level were infected. This accounted for high exposure.

The COVID-19 staff doing duties in laboratory areas and holding areas showed higher antibodies again implying that their work was in a higher risk zone and they needed precautions. Maximum positivity and, hence, the highest level of infection were seen in the respiratory unit and prosthodontics as they were the specialties maximally exposed to infection in COVID-19. Chen WQ et al. performed IgG antibodies to the SARS coronavirus (SARS-CoV) among 1,147 healthcare workers in 3 hospitals that admitted SARS patients in mid-May 2003. They had two groups: one which worked with SARS patients and the other which has never worked with them. Their seroprevalence rate was 88.9% (80/90) for healthcare workers with SARS and 1.4% (15/1,057) for apparently healthy healthcare workers. The seroprevalence in the reference group was 0.4% (3/709) [14]. These findings suggested that inapparent infection was uncommon. Initially, healthcare workers did not realize the importance of precautions in these specialties which may be a contributing factor for their exposure to COVID-19 as indicated by the presence of COVID-19 IgG antibodies. A low level of immunity among unaffected healthcare workers reinforces the need for adequate personal protection and other infection control measures in hospitals to prevent future epidemics.

## 5. Conclusions

Our study shows that the prevalence of detectable antibodies was low in the general population in India and many patients were asymptomatic. The maximum exposure was present in young healthy males of labor class who remained asymptomatic. The healthcare workers were more exposed to COVID-19 as compared to the general population probably due to lack of precaution and awareness. Those working in non-COVID-19 duties were also exposed to an appreciable amount of COVID-19 and needed to take all the precautions required in COVID-19 duties. Rapid kits for IgG can be used for screening purposes.

## Figures and Tables

**Table 1:** COVID-19 IgG positive status among blood donors.

Donor status	No.	%	95% CI for COVID-19 positive
COVID-19 IgG negative	2121	98.1	(1.36–2.52)
COVID-19 IgG positive	42	1.9	
Total	2163	100.0	

**Table 2:** COVID-19 IgG positive status among blood donors according to gender.

Sex	COVID-19 IgG negative		COVID-19 IgG positive		Total		Chi-sq.	*p* value
	No.	%	No.	%	No.	%		
Female	36	1.7	0	0.0	36	1.7	0.725	0.395
Male	2085	98.3	42	100.0	2127	98.3		
Chi-sq.	2121	100.0	42	100.0	2163	100.0		

**Table 3:** COVID-19 IgG positive status among blood donors according to age.

Age	COVID-19 IgG negative		COVID-19 IgG positive		Total		Chi-sq.	*p* value
	No.	%	No.	%	No.	%		
18–29 yrs.	1161	54.7	24	57.1	1185	54.8	2.922	0.404
30–39 yrs.	700	33.0	11	26.2	711	32.9		
40–49 yrs.	221	10.4	7	16.7	228	10.5		
≥ 50 yrs.	39	1.8	0	0.0	39	1.8		
Total	2121	100.0	42	100.0	2163	100.0		

**Table 4:** COVID-19 IgG positive status among blood donors according to the category of blood donation.

*V*/*R*	COVID-19 IgG negative		COVID-19 IgG positive		Total		Chi-sq.	*p* value
	No.	%	No.	%	No.	%		
*F*	471	22.2	12	28.6	483	22.3	58.8	<0.001
*R*	1641	77.4	26	61.9	1667	77.1		
*V*	9	0.4	4	9.5	13	0.6		
Total	2121	100.0	42	100.0	2163	100.0		

*F* = family donors; *V* = voluntary donors; *R* = replacement donors.

**Table 5:** COVID-19 IgG positive status among blood donors according to Hb status.

Group	*N*	Hb				*t* value	*p* value
		Mean	SD	Min.	Max.		
COVID-19 IgG negative	2120	14.19	2.83	8.60	84.00	0.323	0.747
COVID-19 IgG positive	42	14.05	1.33	12.50	17.70		
Total	2162	14.19	2.81	8.60	84.00		

**Table 6:** Distribution of occupation among blood donors with COVID-19 IgG positive status.

Occupation	No.	%
Agriculture	2	4.8
Business	1	2.4
Driver	1	2.4
Labour	4	9.5
Service	2	4.8
Not specified	32	76.2
Total	42	100.0

**Table 7:** COVID-19 antibody status among healthcare workers.

COVID-19 IgG	No.	%	95% CI of COVID-19 IgG positive
Positive	13	2.3	(1.07–3.57) %
Negative	547	97.7	
Total	560	100.0	

**Table 8:** IgG positive amongst different COVID-19 duty areas.

Ward	Total (*N* = 114)	Positive (*N* = 3)		Negative (*N* = 111)		Chi-sq.	*p* value
		No.	%	No.	%		
Triage	19	0	0.0	19	100.0	0.62	0.432
Isolation	41	0	0.0	41	100.0	1.73	0.188
Holding	21	1	4.8	20	95.2	0.46	0.500
Laboratories	35	2	5.7	33	94.3	1.87	0.171

**Table 9:** Comparison of COVID-19 IgG between non-COVID-19 emergencies and COVID-19 ward healthcare workers.

Ward	Total (*N* = 560)	Positive (*N* = 13)		Negative (*N* = 111)		Chi-sq.	*p* value
		No.	%	No.	%		
Non-COVID-19	446	10	2.2%	436	97.8%	0.06	0.805
COVID-19	114	3	2.7%	111	97.3%		

**Table 10:** Specialty-wise distribution of healthcare workers.

Specialty of healthcare workers	% antibody positivity
Respiratory ICU	100
Prosthodontics	100
Trauma surgery	12.5
Blood bank	9.5
Radiodiagnosis	9.1
Plastic surgery	3.7
Urology	3.6
General surgery	3.1
Neurosurgery	2.6

## Data Availability

All data and material were part of the normal work of the university during the COVID-19 pandemic.
